# Stepping Stones Triple P: the importance of putting the findings into context

**DOI:** 10.1186/s12916-014-0260-9

**Published:** 2015-02-04

**Authors:** Cassandra L Tellegen, Kate Sofronoff

**Affiliations:** Parenting and Family Support Centre, The University of Queensland, St Lucia, QLD 4072 Australia; School of Psychology, The University of Queensland, St Lucia, QLD 4072 Australia

**Keywords:** Commentary, Evidence, Stepping Stones Triple P

## Abstract

The Stepping Stones Triple P (SSTP) parenting program is an evidence-based program for parents of children with a disability. A trial of SSTP was recently published in *BMC Medicine*, which reported results of a randomized controlled trial comparing SSTP to care-as-usual. Although the paper described what should be an important replication trial of SSTP, there are significant shortcomings to the scientific approach of the reporting that need to be addressed. The paper initially cites only a few published SSTP studies and describes evidence for the efficacy of the program as “very scarce”. A meta-analysis of studies evaluating SSTP published prior to submission of this paper was not cited. The results are inconsistent with previous evidence for SSTP, yet the authors provide scant interpretation for this inconsistency. Similarly, the unusually high dropout rate of 49% was not adequately explained. The claims that previous research has only been conducted by the developers, has not included children with intellectual disability, and has not used care-as-usual comparison groups, are inaccurate. This commentary explores these issues further in order to place the findings from the recent trial into context.

Please see related article: http://www.biomedcentral.com/1741-7015/12/191.

## Background

Stepping Stones Triple P (SSTP) is an evidence-based parenting program for parents of a child with a disability. The system of programs available includes brief “light touch” versions as well as more intensive group and individual programs. All programs have been subjected to evaluation in randomized controlled trials (RCTs). Kleefman, Jansen, Stewart, and Reijneveld recently published a paper in this journal describing an RCT evaluating the SSTP in a population of parents of children with borderline to mild intellectual disability in the Netherlands [1]. The authors should be applauded for conducting an independent replication trial of an existing parenting program within a specific population, and in a new country. However, there are some important concerns to be raised about this paper. Firstly, the authors present a rationale for conducting the study that does not accurately represent the current state of evidence for SSTP. Secondly, the authors present an impoverished interpretation of the findings within the paper. The lack of long term effects and very high dropout rate were inconsistent with previous SSTP trials, and require proper consideration. This commentary addresses the misrepresentation of the evidence base for SSTP and highlights concerns around the interpretation of findings reported in this recent trial.

### Representation of evidence for SSTP

**Figure 1:**
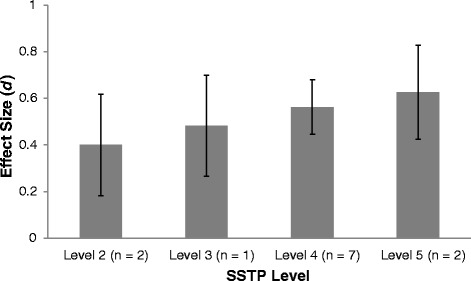
**Effect sizes for different levels of SSTP interventions based on data from the SSTP meta-analysis** **[** 2 **]** **.** *d*, standardized difference effect size; n, number of trials; SSTP, Stepping Stones Triple P-Positive Parenting Program.

The authors describe the previous research on SSTP as being ‘weak’ or ‘very scarce’, stating: “*Although SSTP seems promising, evidence of its effectiveness is very scarce*” [1]. While the authors use the term ‘effectiveness’ in this sentence, they seem to be referring instead to efficacy research and therefore this comment will be interpreted accordingly. The authors reference only four RCTs evaluating the efficacy of SSTP programs and one uncontrolled study. At the time of submission, there were numerous other published and unpublished trials. A more accurate representation of the current evidence base would have cited the SSTP meta-analysis published in 2013, which included 12 studies combining data from 659 families [2]. Figure 1 displays a summary of the effect sizes from the SSTP meta-analysis [2] on child problem data for the different levels of SSTP interventions. In total, there have been nine published RCTs evaluating SSTP, and only four of these were selected for citation. According to standard guidelines, two rigorous RCTs with significant short- and long-term effects are needed for an intervention to be considered efficacious [3]. It is clear that SSTP meets the criteria for an efficacious intervention, and to describe the evidence as ‘very scarce’ is a significant misrepresentation.

In further descriptions of SSTP evidence, the authors state that “*the Australian developers were involved in all the effectiveness studies*” [1]. However, the authors fail to acknowledge that it is typical in intervention research for initial efficacy trials to be developer led, followed by further replication trials led by independent researchers. This is the case for SSTP, with replication trials conducted independently in Australia [4], Japan [5], Germany [6-9], and Canada [10]. The authors made a series of further incorrect assertions: that previous research did not include children with borderline to mild intellectual disability, when trials have included parents of children with intellectual disability (e.g., [11-14]); that previous studies did not compare effects with a care-as-usual (CAU) group, when trials have been conducted with CAU comparisons [4,15]; and that previous studies had small sample sizes when the majority of trials used power analyses to determine appropriate sample sizes.

### Interpretation of findings

The authors reported that, while there were some short-term effects for the intervention, there were no long-term effects for SSTP compared to CAU. This is inconsistent with the results of previous research on SSTP [2,4-9,11-15]; however, the authors make little attempt to explain this inconsistency. For example, an examination of the mean scores on the measures suggests that the lack of long-term effects might be explained by parents in the CAU group continuing to improve over the follow-up period. In contrast, the parents in the intervention group maintained improvements that were seen at short-term; hence, both groups show some improvement. It would not be surprising that parents in the CAU group continued to improve given the large number of parents who received parenting support in that condition. Further exploration of these results could suggest a different interpretation of the effectiveness of the intervention.

The study had an unusually high dropout rate of 49% in the SSTP group. This dropout rate is considerably higher than previous RCTs on SSTP. The average rate of attrition in the intervention group from available data in 11 studies in the SSTP meta-analysis was 13.7% [2]. The authors do not attempt to explain why the dropout rate is much higher in this study except to suggest that the intervention may not fit this population. There is little explanation of why this should be the case or in what respects these families differ from families who have participated in other evaluations of SSTP. Moreover, there was no information provided on fidelity or program adherence for those who did participate, which is standard protocol in intervention research.

It is reported that 34 participants “*did not start the intervention after the intake*” [1], and of the 54 parents that did not complete at least five sessions of SSTP, 46% started another parenting intervention. The main reasons for dropout included starting a comparable parenting support intervention, expectations that the intervention would be too intensive, lack of time, or parents’ non-recognition of child’s psychosocial problems. These reported reasons lead the reader to question the appropriateness of offering this intervention to this population in the way that it was done. The authors provide inadequate information about randomization other than to say that families were blind to condition. What were families told about the programs that they might receive and how were families prepared for an intervention? SSTP is a system of interventions and it could well have been more appropriate to offer some parents a lower intensity level of intervention (such as a Level 2 SSTP parenting seminar, or one or more Level 3 SSTP brief individual consultation sessions [16,17]).

## Conclusions

The results from the recent SSTP trial reported in *BMC Medicine* need to be interpreted in the context of the issues identified with reporting. The recent SSTP trial provided a misrepresentation of the current evidence base for SSTP and failed to fully explore the reasons for the inconsistency of the findings with previous research. It is imperative that future trials of SSTP are conducted following clear and transparent protocols, providing fidelity reports of program delivery, and managing the issue of dropout that seems to have befallen this trial.

In the interests of maintaining scientific integrity, it is also important to acknowledge that a null finding requires replication to the same extent as a positive finding.

## Abbreviations

CAU: Care-as-usual
RCT: Randomized controlled trial
SSTP: Stepping Stones Triple P
